# Evaluation of factors inducing variability of faecal nutrients in captive red deer under variable demands

**DOI:** 10.1038/s41598-021-81908-y

**Published:** 2021-01-27

**Authors:** Stipan Čupić, Andrés J. García, Michaela Holá, Francisco Ceacero

**Affiliations:** 1 Department of Game Management and Wildlife Biology, Faculty of Forestry and Wood Sciences, Czech University of Life Sciences, Prague, Czech Republic; 2 Department of Science and Agroforestry Technology and Genetics, ETSIAM, University of Castilla-La Mancha, Albacete, Spain; 3 Department of Animal Science and Food Processing, Faculty of Tropical AgriSciences, Czech University of Life Sciences, Prague, Czech Republic

**Keywords:** Animal physiology, Biological techniques, Zoology

## Abstract

Based on the assumption that dietary and faecal nitrogen correlate, the number of studies using faecal samples collected in the wild to understand diet selection by wild herbivores and other ecological patterns has been growing during the last years, especially due to the recent development of cheap tools for analysis of nutrients like Near-Infrared Reflectance Spectroscopy (NIRS). Within the annual reproductive cycle, cervids (members of the family Cervidae) face strong seasonal variations in nutritional demands, different for hinds (gestation and lactation) and stags (antler growth) and reflected in differential patterns of seasonal diet selection. In this study we aimed to quantify how pasture availability, season and individual factors like sex, age, reproductive status, body mass and body condition affect faecal nutrients in captive red deer with the goal of understanding how these factors may influence the interpretation of results from samples obtained in the wild with little or no information about the animals who dropped those faeces. We used NIRS for analysing nitrogen, neutral and acid detergent fibres in faeces. The relative influence of some individual factors like pregnancy was low (around 4%), while age and weight may induce a variability up to 18%. The presence or absence of pasture contributed to a variability around 13%, while the season contributed to an average variability around 17% (and up to 21% in certain situations). This high variability in faecal nutrients was observed in a controlled setting with captive animals and controlled diets. Thus, in natural situations we suspect that there would be even greater variation. According to the results, we recommend that preliminary research with captive animals of the species of interest should be conducted before collecting samples in the wild, which should help in the interpretation of results.

## Introduction

Faecal samples are commonly used to estimate the nutritional value of the diet^1^. The nutritional composition of feces is related to the quality of the ingested diet^2,3^, and thus faecal N is commonly used as an index of dietary quality in nutritional ecology studies^4,5^. On the other hand, knowing the nutritional value of the available food, the analysis of faecal samples may reveal the digestive efficiency of an animal; *i.e.*, sometimes high fN may not mean high diet quality but low digestive efficiency. For example, low quality diet may wash down the rumen microbiome and lead to a higher faecal. However, this fact is surprisingly not considered in most studies. The content of neutral detergent fibre (NDF) in forage and faeces may bring some light about it, since fibre fractions inform about feed quality of ungulates^6^ and influences their performance^7^. However, in studies in the wild researchers often have little or no information about the individuals who deposited a sample, and sources of variability related to it. Moreover, food quality and availability are constantly changing, which makes difficult to accurately interpret faecal nutrient values. On the contrary, working with semi-captive or captive animals ensures that diets are controlled, and variations of feed quality are less pronounced, allowing to study the confounding factors affecting faecal nutrients. Furthermore, handling and collection of accurate individual data is easier.

The red deer (*Cervus elaphus*) is a versatile feeder in Mediterranean habitats^8^, with grasses being commonly consumed when the availability is high from late autumn to early summer. In southern Europe during the summer, when grasses are scarce, deer mostly become browser^9–13^. Different seasonal nutritional demands by hinds and stags (driven by different physiological needs) also affect the differential seasonal use of resources between sexes, as well as by young animals^14,15^.

The species is also a seasonal breeder. Hinds enter oestrus between September and February^16^. Gestation and lactation place the greatest nutritional demands on females^17^, and thus, births occur in the period with greater food availability^18^. During this period, growing mothers (often primiparous ones^19^) have higher nutritional requirements since they have to invest energy both for reproduction and for their own growth. Births occur around May and lactation demands are maximum during the first month, decreasing thereafter and becoming low around September^20^ when the new reproductive cycle starts. In males, antler growth is nutritionally expensive^21^ with increased nitrogen demands^22^. However, the period of high nutritional demands in males (early stages of antler development) is not synchronized with that of females (gestation and lactation).

This study aims to examine how the faecal nutrients vary in captive Iberian red deer with very similar feeding regime along the seasons (connected to their annual reproductive cycle and physiological status), and how sex, age, reproductive status, pasture availability, and body traits affect the observed seasonal variations in faecal nutrients, in order to highlight the variability explained by these factors and discuss the adequacy of the technique in studies in the wild when this information is missing.

## Materials and methods

This study was carried out at the experimental deer research facilities of the University of Castilla-La Mancha in Albacete (Spain) during four sampling trials (thereafter *Seasons*) distributed along the reproductive cycle of the species: February (late gestation), May (births), July (mid-lactation) and September (late lactation and rut), during 2017. Forty-three calves and yearlings, 23 stags ranging from 3 to 7 years of age, and 30 hinds were studied, out of which 22 were pregnant. The hinds ranged between 3 and 21 years of age, and were divided into three categories: subadults (aged < 4 at mating; n = 11), adults (aged 4–15 at mating; n = 12), and senescent (aged > 15 at mating, n = 7). All the animals used in this experiment (except one founder female) were born in captivity. Animals were kept in seven 1-ha paddocks, either with bare soil or with irrigated pasture based on tall fescue (*Festuca arundinacea*, 52.4%), cocksfoot (*Dactylis glomerata*, 28.6%), lucerne (*Medicago sativa*, 14.3%), and white clover (*Trifolium repens*, 4.8%). All paddocks used were similar in size and kept a similar (low) density of animals. Irrigation and adequate densities ensured that the pasture quality varied insignificantly along seasons. Animals were assigned to different paddocks and rotated according to management needs (*i.e.*, a given animal could be in a pasture with paddock in one season, but in one with bare soil in another season). All animals were ad libitum fed a Total Mixed Ration (TMR), in order to avoid strong competition for feed^23^. TMR tried to imitate the transition of food in natural conditions, and the ratio of feedstuffs included in the TMR (based on oats, barley, alfalfa meal, cereal straw and citrus pulp) was changing according to the animals’ different nutritional requirements along the year. Stags were also supplemented during all seasons except September with a small daily amount of high protein pellets. Water was provided ad libitum for all animals.

### Data collection and processing

Faecal samples were collected directly from the rectum during routine handling in indoor handling premises^24^ and afterwards stored in labelled paper bags. Routine handling occurs every week, being every animal handled every second week. Thus, samples from each studied animal were collected within two handlings (*i.e.*, all animals were samples within 7 days during each season). Simultaneously, body mass and body condition score^25^ were recorded. In order to achieve uniformity of the faecal samples, they were dried to a constant weight in a hot air dryer at 40 °C for 48 h. Afterwards, they were ground with a mill to pass through a 1-*mm* sieve. Thereafter the samples were thoroughly mixed to achieve maximum homogeneity and dispensed into sample cups for analysis through near-infrared reflectance spectroscopy (NIRS). Four subsamples of the TMR, and feed pellets were obtained for each season. Feed samples were similarly ground (but not dried) and frozen until further analysis (n = 4 for each feedstuff and season).

All the samples were scanned with the NIRS DS 2500 FOSS analyser under the ISIscan Routine Analysis Software (Foss, Hillerød, Denmark). For studies based on faecal samples, an extensive sampling of feed and faeces is inevitably needed. Traditional wet-chemistry methods are time-consuming and expensive, and thus, NIRS technology has become a widely used method that overcomes these drawbacks and allows rapid, low-cost, chemical-free, and non-destructive analysis of a large number of samples^26^. Thus, the technique is already commonly used for measuring food quality through faecal indices in ungulates^1,27,28^. The analyser automatically calculates the average of 8 successive scans at a resolution of 0.5 nm which gives the spectrum of each sample, recorded as the logarithm of the reciprocal of reflectance (amount of radiation reflected from the sample^29^). The software displays the curve of the reciprocal logarithm of reflectance and a curve of absorbing component in a close-to-linear relationship. The peak values of the two curves occur at wavelengths that correspond to absorption bands in the sample (*i.e.,* lower reflectance^30,31^). By this method, the contents of fN (faecal Nitrogen), fNDF (faecal Neutral Detergent Fibre), and fADF (faecal Acid Detergent Fibre) were calculated with WinISI 4 Calibration Software (Foss, Hillerød, Denmark) according to a calibration set previously developed for red deer faecal samples (^32^ based on 100 samples, and showing very high predictive power R^2^ > 0.98). TMR calibration from the *Ruminant Feed Package* (Foss, Hillerød, Denmark) was used for every set of subsamples, and mean value was calculated. All the values obtained with these calibrations showed adequate GH (distance to the population average) and NH (distance to the closest sample) values.

Protein, ADF and NDF in pellets for stags were analysed through standard wet chemistry methods in an specialized lab^33^. Pasture was not collected for this study, since it has been analysed for several previous studies in the experimental facilities. The nutritional composition of pasture (average from previous analyses), TMR, and pellets is shown in Table 1.

**Table 1:** Nutritional composition (dry matter) of the supplemented total mixed ration (TMR), feed pellets and pasture used along the study.

	Pasture	Feed pellets^a^	TMR February	TMR May	TMR July	TMR September
Protein (%)	19	20.264	13.052	16.186	15.791	14.024
Crude Fibre (%)	22	–	–	–	–	–
ADF (%)	–	14.946	43.918	38.896	35.587	43.415
NDF (%)	–	36.879	66.819	58.904	54.291	57.095

^a^Supplemented only to stags during February, May, and July.

Sampling frequencies and handling procedures were designed to reduce the stress of the animals, according to the European and Spanish laws and current guidelines for ethical use of animals in research^34^, and Spanish and European guidelines and laws in the use of animals in research. The research protocols were approved by the Committee of Ethics in Animal Experimentation from the University of Castilla-La Mancha.

### Selected faecal indices

fN is an important parameter for research on diet quality since it shows positive linear relationship with dietary nitrogen^3,35–37^. fN is also stable for few weeks post-defecation in well preserved samples^38^. However, other authors warn about its limitations because the presence of secondary metabolites like tannins decrease protein digestion in ruminants^39,40^, which may lead to misinterpretation of diet quality based on fN.

Fibre fractions (NDF and ADF) are among the faecal constituents that inform about food quality of ungulates^36^. The faecal fibre fractions (fADF and fNDF) are sensitive to fluctuations in food quality, so they should be included to support fN as a proxy for nutritional analyses, especially when diets are expected to contain high amounts of tannins^36^. NDF (which consists predominantly of hemicellulose, cellulose and lignin) reduce voluntary food intake when in high dietary levels. ADF is a subset of NDF which includes the least digestible compounds for herbivores: lignin, cellulose and cutin. As the content of ADF in a diet increases, digestibility and available energy decrease, while, on the other hand, sufficient fibre levels are required in the diet to maintain normal rumen function^6^.

### Statistical analysis

Analyses were performed using IBM SPSS Statistics (version 25.0 for Windows, IBM, USA). Since factors affecting faecal nutrients are different among sex classes, three datasets were created and analysed individually: stags, hinds, and young (calves + yearlings).

General Linear Mixed Models (GLMM; normal distribution with identity link) were used for each dataset. Normality of the variables used for each of the models were previously confirmed by Kolmogorov–Smirnov tests. A data structure based on *ID* as subject and *Season* as repeated measure was used. *Season*, *Age* (young and stags’ datasets), *Age Class* (hinds’ dataset), *Reproductive status* (hinds’ dataset), *Pasture*, *Body Mass*, and *Body Condition Score* entered the model as fixed factors; fN, fNDF and fADF were the response variables of the models (response variables). For hinds it was possible to use both variables *Age* and *Age Class*; thus, preliminary models for the three faecal nutrients were created using the whole set of independent variables plus *Age* or *Age Class*, and the models with *Age Class* had always lower Corrected Akaike’s Information Criterion (AICc). Thus, *Age Class* was used in the models for hinds. Absence of multicollinearity was tested for the independent variables used through the Variance Inflation Factor, which was always low. Initial models were built as described, without including interactions, and were subsequently solved through a stepwise backward selection procedure. Thereafter, a number of interactions were identified as potentially interesting for at least one of the datasets: Reproduction*Age Class, Reproduction*Body Mass, Reproduction*Body Condition Score, Reproduction*Pasture, Reproduction*Season, Season*Age Class, Season*Body Mass, Season*Body Condition Score, Season*Pasture and Season*Sex. Since the sample size did not allow to include all of them at once in the models for any of the datasets, each one was individually tested together with the variables previously described. Only those interactions significant in these preliminary models (*p* < 0.05) were selected for further analyses. For hinds, the selected interactions were: Reproduction*Pasture (*p* = 0.018), Reproduction*Season (*p* = 0.017), Season*Body Mass (*p* < 0.001) and Season*Body Condition Score (*p* = 0.013) for fN; Reproduction*Season (*p* = 0.048), Season*Age Class (*p* = 0.021) and Season*Body Mass (*p* < 0.001) for fNDF; Reproduction*Pasture (*p* = 0.009), Reproduction*Season (*p* = 0.003), Season*Body Mass (*p* = 0.001) and Season*Pasture (*p* = 0.003) for fADF. For males, Season*Age was selected for fN (*p* = 0.004) and fNDF (*p* = 0.003), while no interaction was selected for fADF. For calves, only Season*Sex was selected for fADF (*p* = 0.045), while no interaction was selected for fN and fNDF. New GLMMs were built including these selected interactions, and solved as previously indicated. Thus, for each dataset and response variable three models were built: full initial model without interactions (with no backward selection procedure implemented), solved initial model (with backward selection but without interactions), and solved model including the previously selected interactions. AICc was used for selecting the most plausible models. Thereafter, only the selected models are shown and discussed.

Finally, the coefficients of variation for each faecal nutrient and dataset was calculated in order to understand the variability of the data collected. Thereafter, and considering the mean values for each variable, ranges and the coefficients obtained in the GLMMs, the variability induced by each significant independent variable studied was also calculated for each faecal nutrient and dataset.

## Results

For each dataset (hinds, stags and young), one model was selected for each dependent variable according to AICc (Table 2). The significant variables in the selected models (Table 3) are described below.

**Table 2:** Corrected Akaike´s Information Criterion (AICs) values of each of the three models prepared for each dataset and faecal nutrients (nitrogen—fN; neutral detergent fibre—fNDF; acid detergent fibre—fADF). The selected models, those with lower AICc value, are highlighted in bold. Only these models are described and discussed.

	Hinds	Stags	Young
**fN**			
Initial model	40.678	2.457	− 43.495
Solved model (without interactions)	**33.940**	− **17.858**	− **62.797**
Solved model (with interactions)	37.381	^a^	^a^
**fNDF**			
Initial model	**503.869**	**266.304**	**505.405**
Solved model (without interactions)	531.816	303.811	513.589
Solved model (with interactions)	523.144	280.607	^a^
**fADF**			
Initial model	467.402	**254.428**	508.482
Solved model (without interactions)	481.230	289.930	515.968
Solved model (with interactions)	**453.708**	^a^	**498.698**

^a^The solved model was the same as the solved model without interactions.

**Table 3:** General linear mixed models explaining the content of faecal nutrients in red deer hinds, stags and young (nitrogen—fN; neutral detergent fibre—fNDF; acid detergent fibre—fADF; see Table 2 for more details about the selection process).

	Hinds	Stags	Young
**fN**			
Age	–	F = 8.956, *p* = 0.004**	F = 58.061, *p* < 0.001***
Age class	^ns^	–	–
Sex	–	–	^ns^
Reproduction	F = 5.564, *p* = 0.021*	–	–
Season	F = 42.374, *p* < 0.001***	F = 10.842, *p* < 0.001***	F = 60.695, *p* < 0.001***
Pasture availability	F = 20.697, *p* < 0.001***	F = 4.002, *p* = 0.050*	^ns^
Body mass	F = 13.641, *p* < 0.001***	^ns^	^ns^
Body condition score	^ns^	F = 4.149, *p* = 0.046*	^ns^
Season*age	–	^ns^	–
**fNDF**			
Age	–	F = 5.313, *p* = 0.026*	F = 0.200, *p* = 0.656^ ns^
Age class	F = 3.667, *p* = 0.030*	–	–
Sex	–	–	F = 1.210, *p* = 0.274^ ns^
Reproduction	F = 0.549, *p* = 0.461^ ns^	–	–
Season	F = 14.630, *p* < 0.001***	F = 8.244, *p* < 0.001***	F = 13.380, *p* < 0.001***
Pasture availability	F = 23.604, *p* < 0.001***	F = 0.975, *p* = 0.329^ ns^	F = 6.694, *p* = 0.011*
Body mass	F = 7.098, *p* = 0.009**	F = 3.919, *p* = 0.054^ ns^	F = 0.016, *p* = 0.900^ ns^
Body condition score	F = 2.752, *p* = 0.101^ ns^	F = 0.060, *p* = 0.808^ ns^	F = 0166, *p* = 0.684^ ns^
**fADF**			
Age	–	F = 7.574, *p* = 0.009**	F = 19.294, *p* < 0.001***
Age class	F = 15.446, *p* < 0.001***	–	–
Sex	–	–	^ns^
Reproduction	F = 4.713, *p* = 0.033*	–	–
Season	F = 8.475, *p* < 0.001***	F = 9.642, *p* < 0.001****	F = 25.756, *p* < 0.001***
Pasture availability	^ns^	F = 0.273, *p* = 0.604^ ns^	^ns^
Body mass	^ns^	F = 5.715, *p* = 0.021**	^ns^
Body condition score	^ns^	F = 0.117, *p* = 0.734^ ns^	^ns^
Season*body mass	F = 6.342, *p* < 0.001****	–	–
Season*pasture	F = 4.906, *p* = 0.001**	–	–
Season*sex	–	–	F = 2.463, *p* = 0.050**

Dashes indicate variables not included in a certain model.

^ns^Indicate variables included in a certain model, but not significant.

Statistically significant differences at 0.05, 0.01, and 0.001 are indicated by *, ** and ***, respectively.

### Faecal nutrients in hinds

**Figure 1:**
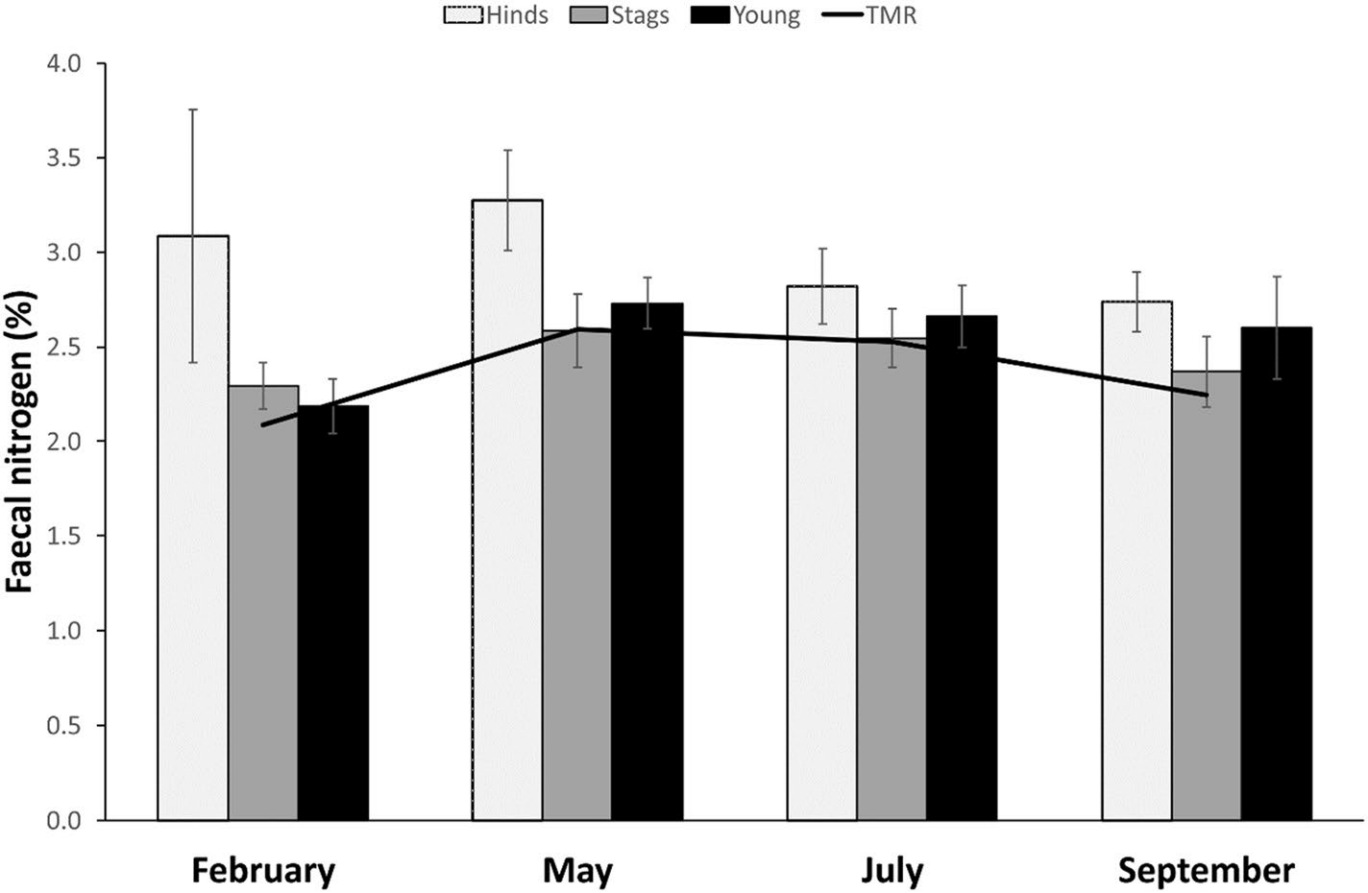
Faecal nitrogen content for red deer hinds, stags, and young along the 4 studied periods (seasons). The line indicates the percentage of nitrogen found in the feedstuff (*TMR* total mixed ration) that animals were provided during the study. Males were supplemented with extra small amounts of 20% protein feed pellets in February, May and July.

The fN was lower in hinds under reproductive constraints (t = -2.359, β =  − 0.119). Hinds living in paddocks with pasture had higher fN (t = 4.549, β = 0.399). Body mass also had a negative effect on fN (t =  − 3.693, β =  − 0.006). Season was an important source of variability (Fig. 1): fN was significantly higher in February compared to July (t = 2.199, β = 0.280, *p* = 0.030), in February compared to September (t = 2.941, β = 0.366, *p* = 0.004), in May compared to July (t = 8.488, β = 0.506, *p* < 0.001), and in May compared to September (t = 11.039, β = 0.592, *p* < 0.001).

**Figure 2:**
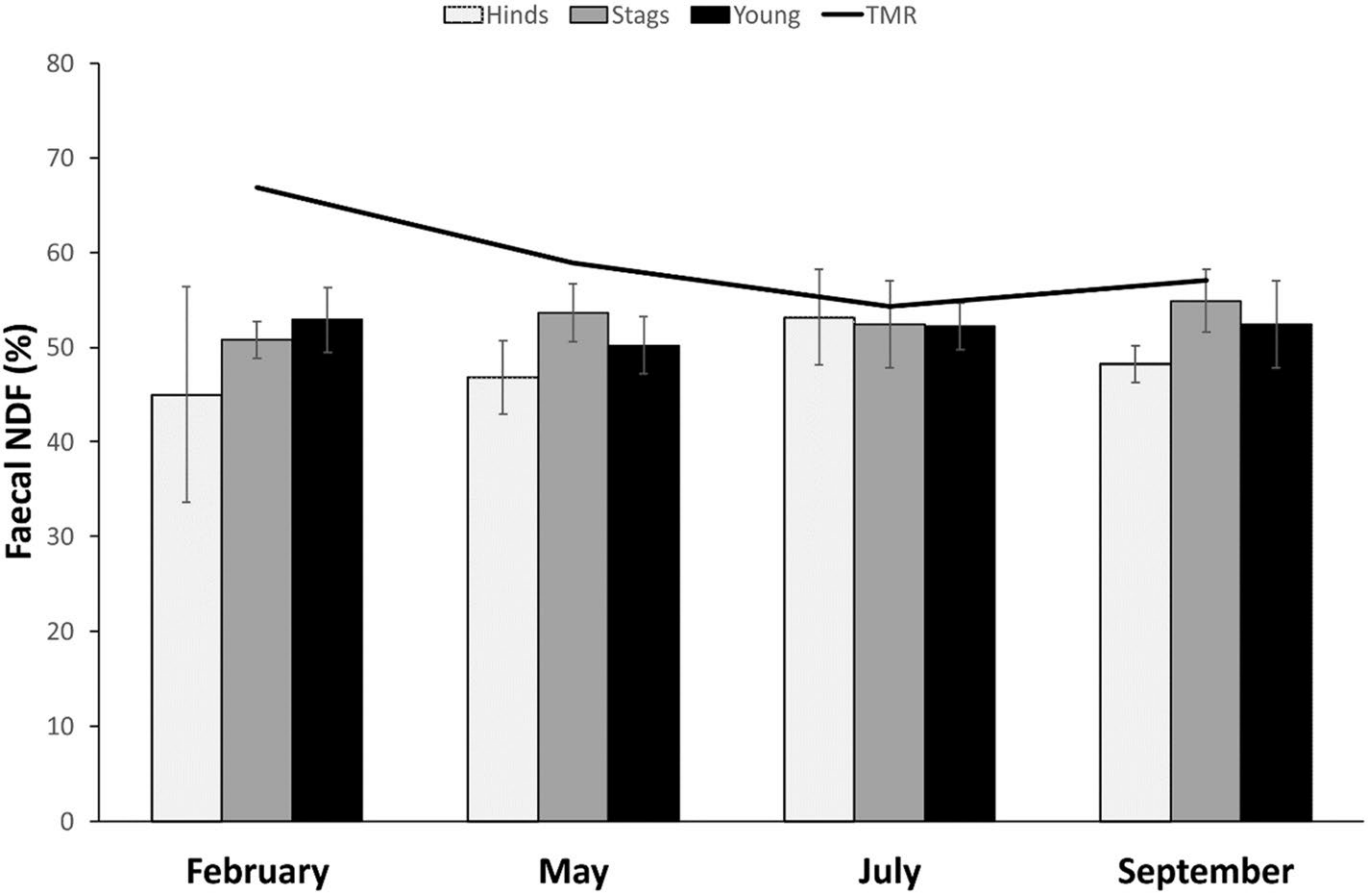
Faecal NDF content for red deer hinds, stags, and young along the 4 studied periods (seasons). The line indicates the percentage of NDF found in the feedstuff (*TMR* total mixed ration) that animals were provided during the study.

The fNDF was higher in heavier hinds (t = 2.664, β = 0.109). Hinds in paddocks with pasture had lower fNDF (t =  − 4.858, β =  − 6.134). Adult hinds had higher fNDF than subadults (t = 2.151, β = 1.342, *p* = 0.034) and senescent (t = 2.453, β = 1.879, *p* = 0.016). Season was also a source of variability (Fig. 2): fNDF was significantly higher in July compared to February (t = 3.846, β = 9.102, *p* < 0.001), in July compared to May (t = 6.086, β = 7.125, *p* < 0.001), in July compared to September (t = 5.415, β = 4.968, *p* < 0.001), and in September compared to May (t = 2.542, β = 2.157, *p* = 0.013).

**Figure 3:**
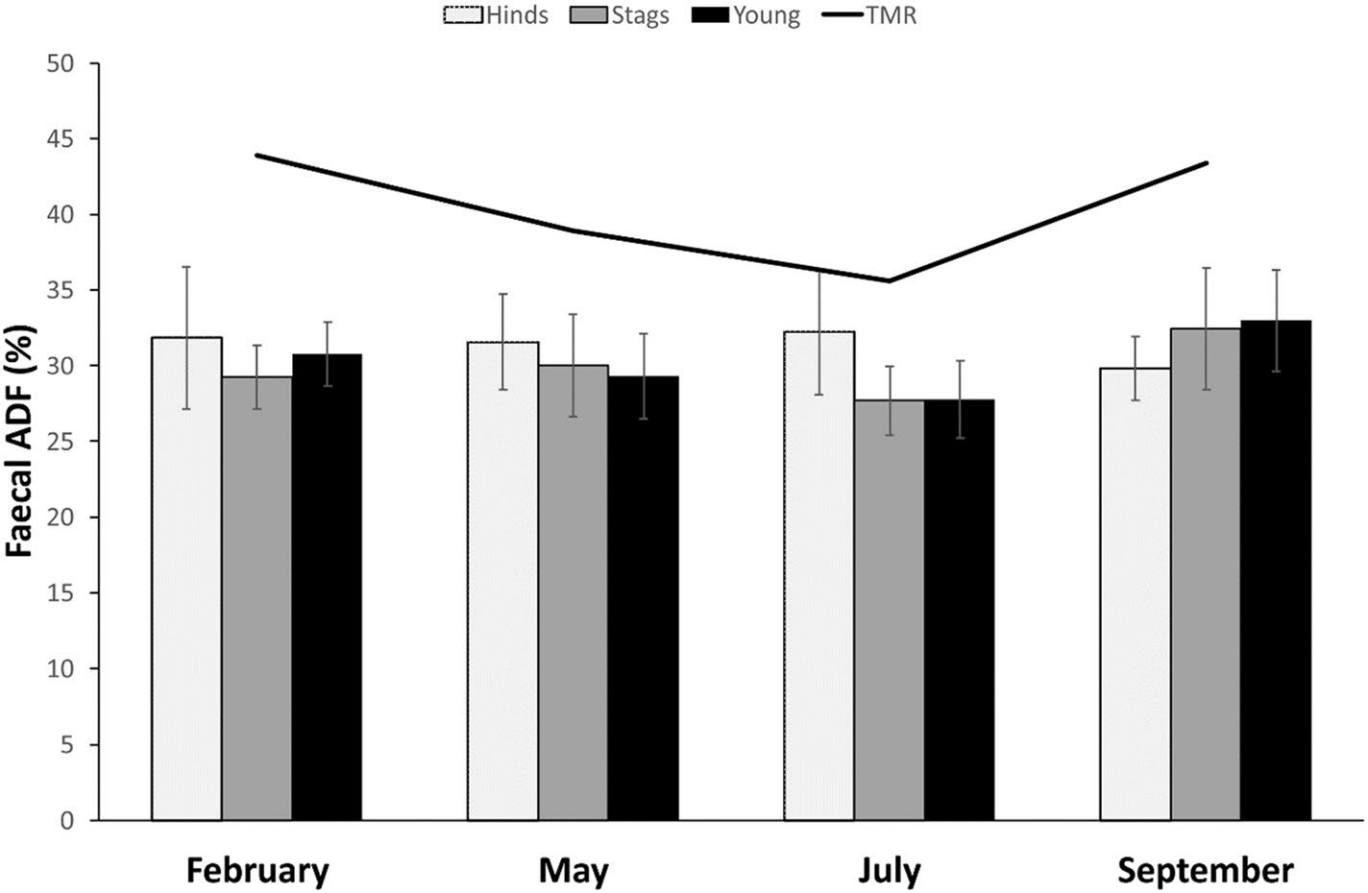
Faecal ADF content for red deer hinds, stags, and young along the 4 studied periods (seasons). The line indicates the percentage of ADF found in the feedstuff (*TMR* total mixed ration) that animals were provided during the study.

The fADF was lower in hinds under reproductive constraints (t =  − 2.171, β =  − 1.392). As for fNDF, adult hinds had higher fADF than subadults (t = 2.123, β = 1.268, *p* = 0.037) and senescent (t = 5.530, β = 4.038, *p* < 0.001), and subadults had higher fADF than senescent (t = 4.008, β = 2.771, *p* < 0.001). Contrary to fN and fNDF, season was a weaker source of variability (Fig. 3): even if it was overall significant, no pairwise comparison was significant itself. This is probably due to the significant interaction Season*Pasture. When the animals were in paddocks with pasture, significantly lower fADF was found in September compared to all the other seasons; on the contrary, in bare-soil paddocks the fADF was higher in September compared to February and May. The interaction Season*Body Mass showed a significant positive correlation of body mass and fADF in February, but a significant negative correlation between them in May.

### Faecal nutrients in stags

The fN was lower in older stags (t =  − 2.993, β =  − 0.051). Stags living in paddocks with pasture had lower fN (t = 2.001, β = 0.193). Body condition had a positive effect on fN (t = 2.037, β = 0.159). Season was also a source of variability (Fig. 1): fN was significantly higher in May compared to February (t = 4.426, β = 0.280, *p* < 0.001), and September (t = 4.494, β = 0.302, *p* < 0.001), and in July compared to February (t = 2.151, β = 0.157, *p* = 0.036), and September (t = 2.888, β = 0.178, *p* = 0.006).

The fNDF was higher in older stags (t = 2.305, β = 1.104) and marginally lower in heavier ones (t =  − 1.980, β =  − 0.066, *p* = 0.054). Season was an important source of variability (Fig. 2): fNDF was significantly higher in September compared to February (t = 4.962, β = 7.544, *p* < 0.001), May (t = 2.467, β = 4.057, *p* = 0.018) and July (t = 2.389, β = 3.538, *p* = 0.021), higher in July compared to February (t = 2.547, β = 4.006, *p* = 0.014), and higher in May compared to February (t = 2.291, β = 3.488, *p* = 0.027).

The fADF was higher in older stags (t = 2.752, β = 1.009, *p* = 0.009) and lower in heavier ones (t =  − 2.391, β =  − 0.068, *p* = 0.021). Season was also a source of variability (Fig. 3): fADF was significantly higher in September compared to February (t = 4.889, β = 7.102, *p* < 0.001), May (t = 3.691, β = 5.248, *p* = 0.001) and July (t = 4.826, β = 5.922, *p* < 0.001).

### Faecal nutrients in young

The fN was higher in calves compared to yearlings (t = 7.620, β = 0.406). Season was also a source of variability (Fig. 1): fN was significantly higher in May compared to February (t = 12.400, β = 0.541, *p* < 0.001) and September (t = 6.740, β = 0.314, *p* < 0.001), in July compared to February (t = 10.081, β = 0.474, *p* < 0.001) and September (t = 4.957, β = 0.247, *p* < 0.001), and in September compared to February (t = 4.718, β = 0.227, *p* < 0.001).

The fNDF was lower in young living in paddock with pasture (t =  − 2.587, β =  − 6.967). Season was an important source of variability (Fig. 2): fNDF was significantly higher in September compared to February (t = 2.872, β = 2.838, *p* = 0.005), May (t = 5.978, β = 5.597, *p* < 0.001) and July (t = 4.355, β = 3.489, *p* < 0.001), in February compared to May (t = 3.131, β = 2.759, *p* = 0.002), and in July compared to May (t = 2.431, β = 2.107, *p* = 0.017).

The fADF was lower in calves than in yearlings (t =  − 4.392, β =  − 4.082, *p* < 0.001). Season was also a source of variability (Fig. 3): fADF was significantly higher in September compared to February (t = 5.076, β = 3.732, *p* < 0.001), May (t = 6.148, β = 5.223, *p* < 0.001) and July (t = 8.511, β = 6.550, *p* < 0.001), and in February compared to July (t = 4.136, β = 2.818, *p* < 0.001).

### Variability induced by the studied factors on the observed faecal nutrients values

The coefficients of variation (c.v.) of the faecal nutrients analysed in hinds, stags and young showed low values (most of them below 15%; Table 4). These were much lower in young than in adults (7.3% *vs*. 12.8% on average), and much higher in February (15.4%) than in the other seasons (9.6%, 9.7%, and 9.2% for May, July and September respectively). On the other hand, fN in hinds and fADF in hinds and stags during February even exceeded a 20% c.v.

**Table 4:** Coefficients of variation (c.v.) of the studied faecal nutrients (nitrogen—fN; neutral detergent fibre—fNDF; acid detergent fibre—fADF) for each dataset (hinds, stags and young) and each sampling period.

	Hinds	Stags	Young
**fN**			
February	21.72	18.64	6.56
May	8.12	13.25	4.97
July	7.09	16.33	6.19
September	5.81	12.60	10.34
**fNDF**			
February	14.76	13.78	6.75
May	10.01	8.87	9.60
July	12.81	10.56	9.15
September	7.08	8.04	10.15
**fADF**			
February	25.34	26.08	4.54
May	8.30	17.41	6.03
July	9.52	11.34	4.62
September	4.03	16.36	8.79

According to the average faecal nutrient values recorded and the coefficients (β) obtained in the models for the significant variables, we calculated the variability induced by them (Table 5). While the relative influence of some factors like pregnancy is low (around 4% variation between pregnant and not pregnant hinds), other individual characteristics like age and weight induce a variability up to 18%; *e.g.*, it can be expected that, even under the same diet, the heavier and the lighter hinds in a population will vary in their fN values with 16%. The presence or absence of pasture induce a variability around 13%, while the season induce a variability around 17% (on average) and in certain situations up to 21%.

**Table 5:** Variability induced by the individual and nutritional factors studied in the faecal nutrients (nitrogen—fN; neutral detergent fibre—fNDF; acid detergent fibre—fADF; see Materials and Methods for more details about the calculations).

	Hinds (%)	Stags (%)	Young (%)
**fN**			
Reproduction	4.0		
Pasture availability	13.4		
Age		10.4	15.8
Weight	16.3	7.9	
Season	19.8	12.3	21.1
**fNDF**			
Age Class	3.9		
Pasture availability	12.7		13.4
Age		10.4	
Weight	18.1	14.2	
Season	18.9	14.2	10.8
**fADF**			
Reproduction	4.4		
Age Class	12.9		
Age		9.5	13.3
Weight	4.0	17.9	
Season	19.3	13.4	21.3

## Discussion

Even under a controlled environment with healthy animals and very similar feeding sources, red deer showed a relatively high variability in the analysed faecal nutrients: fN, fNDF and fADF. This variability jumped in some situations above 25%, and all the individual factors studied (age/age class, body mass, sex, reproductive status, body condition) as well as season and pasture availability significantly contributed to explain such variability in the faecal nutrient content to a certain degree. These results have important implications for the interpretation of ecological studies based on faecal nutrients conducted in the wild, with low or any information about the individuals being sampled.

Faecal N has been widely accepted to correlate with the N content of the diet^35,36^. However, while faecal N appear to mirror dietary N in young individuals (see Fig. 1), this seems to not always be the case for adults. Stags and hinds are known to adopt different feeding strategies which certainly reflect their physiological adaptations, especially during nutritionally demanding periods: while hinds select for quality, stags opt for a greater amount of poorer quality food^41^. In stags, the variation in fN appears to mirror the seasonal variation in forage nitrogen content in all the four sampling periods even if stags were additionally supplemented with small amounts of high protein feed pellets in all seasons except September. This suggests that the extra protein ingestion was not reflected in the fN. However, this mirroring between dietary and fN observed in stags is more complex than it seems at first glance. Because of their larger size, stags have greater total requirements compared to pregnant or lactating hinds^42^. With their mouths, stags can ingest larger, more fibrous food particles with less nitrogen on a percentage dry weight basis, and they compensate this lower-quality food by eating more relative to their size^41^. High fibre diets decrease the digestibility^43^ and increase the retention time^44^, which leads to increased faecal loss of dry matter and N. But also to increased fNDF^43^, which in our study was always higher for stags compared to hinds, except in July (probably due to their high lactation demands and increased selectivity of good quality food). It has been reported that when placed on a poor-quality diet, stags did not increase their body mass during the summer which was in contrast to lactating hinds, despite consuming diets of identical quality^43^. Lower contents of N in rumen of stags in winter compared to those of hinds have also been reported^41^. These are consistent results with ours and support lower concentration of fN in February for stags compared to hinds, despite stags being provided feed pellets rich in protein. In our research, stags on pasture had lower fN which supports the hypothesis of increased intake of low-quality food in order to balance the final N intake^45^. Feed pellets seems to be important source for stags to exceed daily maintenance requirements for protein and energy to replenish body reserves and to compensate for the inability to do so from the lower-quality food which they eat abundantly. Alternative explanation is that extra N ingestion was not reflected in the fN due to a high digestive efficiency at least during the three supplemented periods which match with the antler growth period^46^.

Contrary to males, in hinds fN was always higher than expected based on the amount of dietary N. This result might seem surprising since February and May are periods with highest demands because of reproduction^17,21^, and thus, greater digestive efficiency would be expected. On the other hand, from February to May (late pregnancy) red deer hinds tend to decrease the volume of rumen^47^, which means decreased food intake. This may have led to a greater selectivity for the feedstuff items with greater protein content^23^. Lowest ingestion by hinds from February until the approximate time of parturition has been reported^47^, indicating higher selectivity for good-quality food. After parturition, demands for nutrients associated with lactation rise^48^ which is followed by increased weight and size of the rumen, abomasum, intestines, and liver^49,50^. Large herbivores tend to eat more and increase body mass during the summer months to support breeding energy expenses^43^. Furthermore, after weaning hinds remodel the digestive tract and increase rumination^51^ which may have enhanced their ability to extract nitrogen from their forage. Reindeer have been reported to have increased digestibility and food intake during increased lactation demands^52^. Forages with high levels of digestible energy (like high non-structural carbohydrates) generate high rates of fermentation^36,53^ therefore, high rates of intake and consumption of high-quality forages result in high values of faecal nitrogen via increased fermentation, absorption of microbial biomass, and bypass of nitrogen^6^. Later on, the elevated protein excretion was not pronounced in July and September as much as in previous seasons, which correspond to the period of normal volume of hinds’ rumen^47^.

The difficult interpretation of fN for hinds and stags is mostly associated with the lack of information about concentration of nutrients in rumen. The nutritional value of rumen content would have allowed calculation of undigestible nitrogen that can only be digested by fermentative processes. Analysing N bound to ADF in faecal samples (NDF-N^6^) may be an interesting alternative for future studies. This index allows to calculate metabolic faecal nitrogen (MFN) as fN minus fNDF-N.

Some authors have warned about the influence of secondary metabolites like tannins, which may decrease protein digestibility^39,40^. This factor is unlikely to have affected our results since only common feedstuffs for livestock with low content of plant secondary compounds (PSCs) were used in the study. Other authors have also warned of limitations of fN as dietary index due to adaptations by the deer to relatively poor diets^51^. They showed that lactating hinds have an ability to more thoroughly process the food via mastication, extracting more plant proteins from cell wall surfaces and increasing digestion of finer particles^54^. Also, the ability to remodel the gastrointestinal tract during lactation^50^ contribute to increase N absorption, resulting in additional reduction in N excretion^51^. For these reasons, fN may provide an incorrect impression of the relative quality of the diet. At this stage, it is necessary to highlight that the rumen microbiome and nitrogen recycling into the rumen are factors difficult to control in studies in the wild that may explain the variability in faecal nutrients found in this study (especially fN), but also even the differences in functioning mechanisms among sexes^41^.

Similarly, faecal fibre fractions (fNDF and fADF) are considered to reflect diet quality and to be sensitive to fluctuations in food quality^36^. They are suggested to be analysed in order to support the interpretation of fN, since in studies in the wild it would be difficult to determine the dietary N. Both NDF and ADF reduce voluntary food intake and digestibility^6^. Indeed, as expected, dietary N and fibres in our main feedstuff (TMR) are inversely related in our study (see Figs. 1, 2 and 3). Nevertheless, neither fNDF nor fADF fairly reflected dietary levels: while dietary levels of NDF and ADF varied seasonally, very low variability across seasons and age/sex classes were observed for fNDF and fADF. Similarly to fN, dietary fibres seem to match faecal ones just for calves, but not in adults.

Our results also highlight the influence of several individual factors on fN for each of the age/sex class studied. Further than the previously discussed low fN with high dietary N during antler growth, we found a significant effect of body condition: stags in low condition had lower fN; that is, they were more efficient using the dietary protein. Indeed, we also found a significantly lower fN values in hinds under reproductive constraints (pregnancy or lactation); however, this effect was quite low since we estimated that the variability in fN due to reproductive constraints is just 4% (Table 5). Other studied factors like age also showed sources of variability different for hinds and stags, which further support the previous observations. Age is an important source of variability in stags, around 10% for all fN, fNDF, and fADF, while age class was not a significant factor explaining faecal nutrients in hinds, just around 4% for faecal fibres. In stags, fN was lower in older individuals, which fits with the results previously discussed: while antler investment and requirements increases with age^21,55^, the reproductive investment is relatively constant for hinds, independently of their age.

Our results have important implications for studies using this technique in the wild. Faecal N is commonly used as an index of dietary quality in nutritional ecology studies^4,5^. However, the variability observed for some individual factors like age, body mass, body condition, and reproductive and antler growth constraints can hardly be estimated in faecal samples collected in the wild, and thus may mislead the conclusions drawn from the results obtained without considering these factors. Furthermore, our results also show important influence of the presence of pasture and the season (physiological status). First, as already explained, diet quality determination through faecal fibres in certain situations (if not approached carefully) can be quite inaccurate and induce errors. Moreover, the presence of pasture in our study induced a variability around 13% in fN and fNDF, especially in faecal samples from hinds. The seasonal effect is also evident, being significant for all the studied nutrients and sex/age classes. The variability induced by the season ranged from 10 up to 21% in a set up with relatively stable feeding regime. This fact suggest that one should be cautious when comparing faecal samples from wild animals from different seasons and locations, especially considering the important seasonal variations in the diet selection by the species in the wild^1,9–12,56^. Further consideration is also necessary for the already highlighted fact of increased fN when diets are rich in tannins or another PSCs^40^: these may be much higher in natural diets, increasing the degree of uncertainty of the results obtained.

Our results also confirm that NIRS is a powerful tool to investigate feeding and nutrition of herbivorous ungulates. However, our results clearly suggest that thorough preliminary studies with the target species of interest under controlled conditions are necessary in order to previously validate the technique and determine the degree how multiple factors (mainly linked to individual characteristics) may affect the interpretation of data obtained from samples collected in the wild. That may help for defining sampling strategies in the wild and to interpret the results obtained. Definitely, the results highlight that more studies with captive animals (other taxa) under controlled conditions are needed to evaluate if faecal indices can be used as a proxy for studies in the wild. It would be also interesting to perform a similar study in the same species in different latitudes: in Mediterranean habitats, summer is commonly the season with low feed availability, while it is winter in temperate climates. That may greatly influence the results in experimental settings similar to ours.
